# Unauthorized Horizontal Spread in the Laboratory Environment: The Tactics of Lula, a Temperate Lambdoid Bacteriophage of *Escherichia coli*

**DOI:** 10.1371/journal.pone.0011106

**Published:** 2010-06-14

**Authors:** Ella Rotman, Luciana Amado, Andrei Kuzminov

**Affiliations:** Department of Microbiology, University of Illinois at Urbana-Champaign, Urbana, Illinois, United States of America; University of Massachusetts Medical School, United States of America

## Abstract

We investigated the characteristics of a lambdoid prophage, nicknamed Lula, contaminating *E. coli* strains from several sources, that allowed it to spread horizontally in the laboratory environment. We found that new Lula infections are inconspicuous; at the same time, Lula lysogens carry unusually high titers of the phage in their cultures, making them extremely infectious. In addition, Lula prophage interferes with P1 phage development and induces its own lytic development in response to P1 infection, turning P1 transduction into an efficient vehicle of Lula spread. Thus, using Lula prophage as a model, we reveal the following principles of survival and reproduction in the laboratory environment: 1) stealth (via laboratory material commensality), 2) stability (via resistance to specific protocols), 3) infectivity (via covert yet aggressive productivity and laboratory protocol hitchhiking). Lula, which turned out to be identical to bacteriophage phi80, also provides an insight into a surprising persistence of T1-like contamination in BAC libraries.

## Introduction

Organisms are adapted to their natural environments by the fact that they can multiply there and settle in new niches, demonstrating the ability to secure resources (survival), to leave progeny (reproduction) and to find new habitats (spread). The laboratory environment is unique in that it denies organisms control of growth, multiplication and spread: they can do so only *when* they are allowed to, and to the *extent* they are allowed to, by the experimenter. Because of the uniformity of laboratory cultures, contamination in the form of co-habitation is usually detectable and preventable, unless the co-habitant looks exactly like the experimental material. Thus, even though contamination in the laboratory does happen on a regular basis, it does not lead to subsequent horizontal spread of the contaminants, due to differences in appearance, growth cues and protocol-inherent barriers. Yet, the few examples of horizontal spread in the laboratory environment confirm the existence of strategies to overcome human-imposed control over homogeneity, multiplication and cross-contamination. Cross-contamination of clinical samples with the positive control strains of pathogenic bacteria is a well-known, if under-appreciated, challenge for clinical laboratories [1], [2], . However, “being chosen as a positive control” is not a spreading strategy for pathogens in the laboratory and only indicates the ease of cross-contamination. The notorious levels of cross-contamination of various vertebrate cell lines with HeLa cells [4] highlight the survival strategy based on mimicry: by looking like the experimental material, yet multiplying faster and spreading through aerosol, HeLa cells mastered survival via horizontal spread in the laboratory environment, becoming the “weed of cell cultures” [5]. Mycoplasma cross-contamination of cultured eukaryotic cells may be an example of another successful strategy, based on commensalism and, again aerosol spreading [6], although in this case the sheer number of different mycoplasma species that are found as contaminants argues against specific adaptation to horizontal spread. At the same time, no persistent contamination of bacterial laboratory strains with commensals, like prophages, has been documented in scientific publications.

While characterizing sensitivity of *E. coli* Δ*ligB* strain to various DNA damaging agents, we eventually realized that the DNA damage sensitivity was, in fact, not due to the *ligB* deletion, but to a resident prophage of an unknown temperate bacteriophage, which we called “Lula”. The majority of bacteriophages are strictly lytic, in that they propagate by killing and consuming their host, but a minority of bacteriophages, called “temperate” phages, are capable of switching from the lytic mode into a dormant mode, called lysogeny, during which their genomes are passively replicated as “prophages” by the otherwise normal host cells, called “lysogens”. Reports of DNA damage sensitivity due to resident prophages was a common occurrence in 1960s [7], [8], — but an unknown prophage would be unexpected in the “cleansed” laboratory backgrounds in common use these days. We then discovered that quite a few clones in our laboratory collection were contaminated with this commensal, attesting to its spreading powers. However, only when we found that some newly-arriving strains from the *E. coli* Genetic Stock Center or from other laboratories were also infected with the exact same prophage, did we comprehend both the scope of the infection and the uniqueness of Lula.

We realized that, for such an apparent ease of infection, this bacteriophage must use specific tactics to facilitate its spread from strain to strain under laboratory conditions, — in essence, it survives and multiplies in the laboratory environment, its own ecological niche. Periodic assaults on large-scale microbial fermentation by lytic phages is a problem in commercial microbiology, but these nuisance phage infections are effectively self-limiting by lysis, with no persistence or cross-contamination [9]. Perhaps the only known example of a bacteriophage successfully spreading among laboratory cultures is the continuing contamination of BAC libraries with the supposedly lytic T1-like phages [10], [11], [12]. Since lytic phages completely kill their host, the several decade-long persistence of a “T1 relative” in the laboratory setting is a mystery. We decided to characterize specific traits of Lula enhancing its survival in the laboratory, especially those that increase its infectivity, if only to better control contamination with this bacteriophage in the future and maybe to understand the enigma of persistent T1-like contamination.

## Results

### Revealing Lula contamination

**Figure 1:**
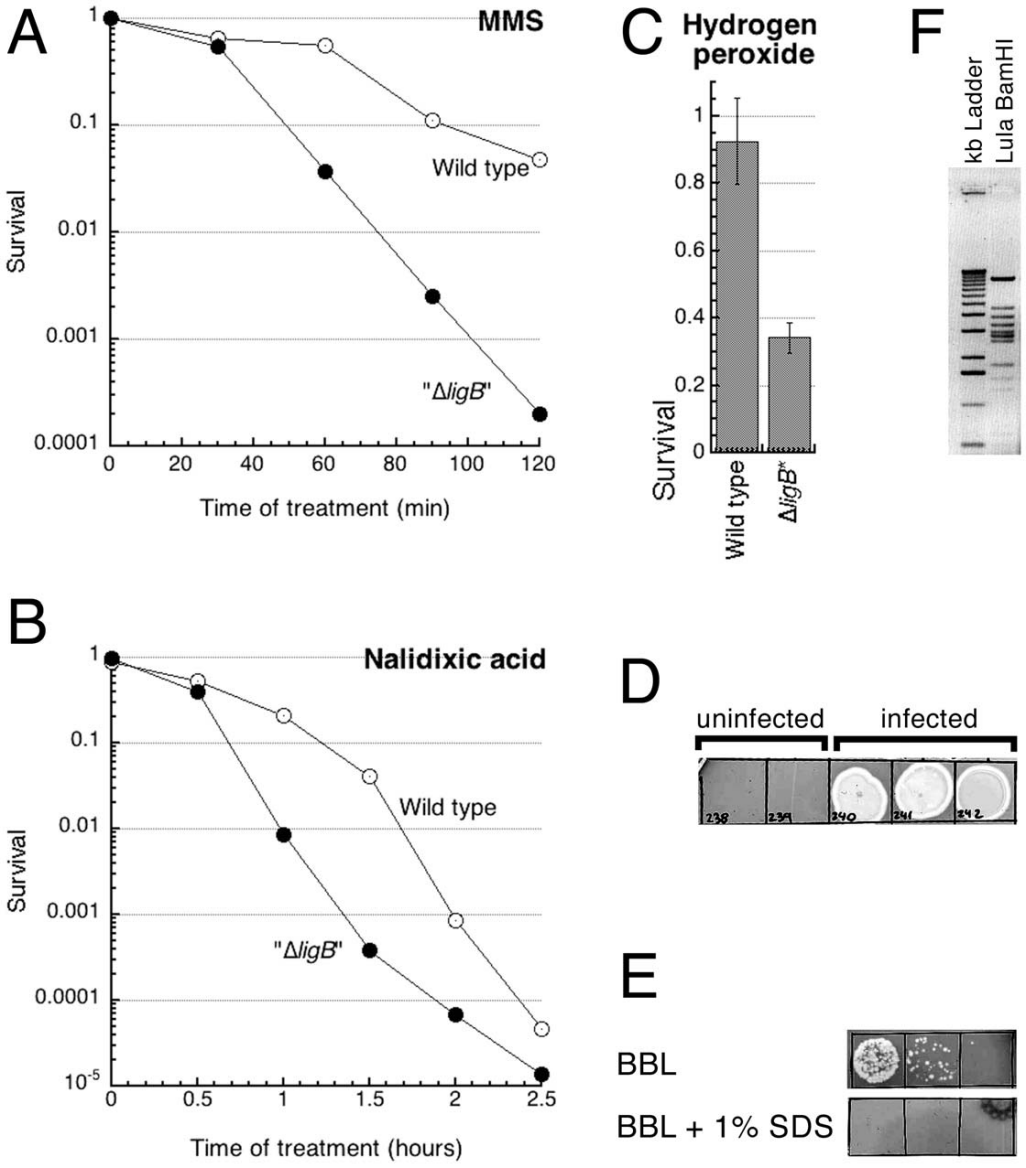
The DNA-damage sensitivity of the “Δ*ligB*” mutants and the assay for Lula presence. **A.** MMS treatment. Strains: wild type, GR523; “Δ*ligB*”, LAP1. **B.** Nalidixic acid treatment. Strains as in “A”. **C.** Hydrogen peroxide treatment. Strains as in “A”. **D.** Assay for the presence of Lula. Supernatants of saturated cultures were spotted by 10 µl onto a lawn of uninfected cells (AB1157), and the plates were incubated at 30°C for 20 hours. **E.** SDS-sensitivity of Lula. 10 µl of the first, second and third dilution of a supernatant of saturated lysogen culture were spotted on a lawn of uninfected cells (AB1157). **F.** An inverted image of ethidium bromide-stained gel showing Lula virion DNA digested with BamHI.

While characterizing what appeared as a considerable DNA damage sensitivity of the Δ*ligB* mutant (Fig. 1A–C), we noticed occasional signs of a limited lysis in the parallel wild type culture, suggesting bacteriophage contamination. Investigating this contamination, we found that: 1) it was coming from the Δ*ligB* mutant, which was apparently harboring a prophage of a temperate bacteriophage; 2) that the DNA damage sensitivity of the original Δ*ligB* mutant was actually separable from the Δ*ligB* defect by P1 transduction and was fully due to the prophage. Realizing that transmission of this prophage, which we subsequently called “Lula”, was happening *during* P1 transduction, we eventually identified the source strain and some other infected strains in our collections. In all cases, the presence of Lula was associated with an increased sensitivity to DNA damaging agents, unless the strain was already sensitive due to other DNA metabolism defects (see below).

Since Lula accumulates to high culture titers during normal growth of a lysogen (see below), the easiest assay to identify Lula contamination is to grow the suspected strain to saturation, to remove cells by centrifugation and to spot 10 µl of the supernatant on a lawn of a non-lysogen (Fig. 1D). Contamination of P1 lysates with Lula can be similarly checked by spotting them on a lawn of non-lysogen, supplemented with 20 mM sodium citrate to inhibit growth of P1. We also noticed that the frequency of contamination with Lula during P1 transduction decreases as the temperature of the subsequent outgrowth increases. In fact, P1 transduction using contaminated lysates at 42°C yields mostly Lula-free transductants and can be used to prevent Lula infection.

Lula is extremely stable as a prophage. We tried to cure it by passaging lysogens at 42°C and/or on LB supplemented with 1% SDS (this completely prevents infection with any phage, including Lula (Fig. 1E)) and checking individual colonies for Lula presence, but found none that became Lula-free. We also tried UV-irradiating lysogen cultures on plates, looking for Lula-free survivors, but even when the original titer was reduced five orders of magnitude by irradiation, there were no non-lysogens among dozens of survivors we checked (we were screening for wild type UV resistance).

### Lula interaction with other phages

The pattern of BamHI digestion of Lula virion DNA (Fig. 1F) did not match the BamHI digestion pattern of Lambda, the textbook temperate phage of *E. coli*, indicating that Lula is a completely different phage. From the fragment sizes, the total size of Lula genome was estimated to be at least 40 kbp, which is similar to the Lambda genome (48,502 bp). Sequencing of the ends of several cloned BamHI fragments revealed no homology to existing database entries (demonstrating that Lula is an unpublished phage, — it was at this point it has gotten its own name), yet similarities to N15, HK97 and *Salmonella* Gifsy phages, suggesting that Lula is a lambdoid phage.

**Figure 2:**
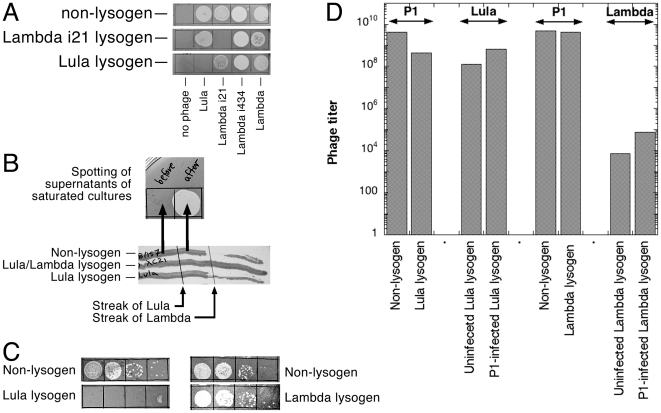
Interaction with Lambda, T4 and P1 phages. **A.** Plating of Lula and various Lambdas on each other's lysogens. Strains are: non-lysogen, AB1157; Lambda i21 lysogen, MO (λi^21^); Lula lysogen, EL103. **B.** The lysogeny test. First, fresh colonies of a non-lysogen (AB1157), a Lula single lysogen (EL103) and a Lula/lambda double lysogen (MO (λi^21^)(phi80)(λ *vir* ^R^)) are streaked horizontally from left to right across two vertical phage lines — the left one made with a high-titer stock of Lula, the right one made with a high-titer stock of Lambda. The next day, since the non-lysogen grew equally well both before and after crossing the Lula streak, we took cells from indicated locations, streaked them to single colonies and passed these clones through “Lula contamination” test (Fig. 1D). The test confirmed that, although no lysis is apparent, cells become Lula lysogens after crossing the Lula line. **C.** Lula lysogens do not plate T4. Serial dilutions of T4 stock were spotted by 10 µl on lawns of either a non-lysogen (AB1157), Lula lysogen (EL103) or Lambda lysogen (EL104). **D.** Interaction of Lula and Lambda lysogens with P1. The P1 columns: P1 lysate was prepared in parallel on the two cultures of the same density, and the resulting P1 phage titer was determined either at 42°C (to inhibit Lula) or on a Lambda lysogen (to inhibit Lambda). “Lula” or “Lambda” columns: either mock-infected or P1-infected corresponding lysogen was taken through the “preparation of P1 lysate” procedure, and the titer of the phage was determined in the resulting lysate by plating in the presence of 20 mM Sodium Citrate (to inhibit P1). The values are averages of two measurements. Strains are: non-lysogen, AB1157; Lula lysogen, EL103; Lambda lysogen, EL104.

A Lula lysogen plates Lambda at the same titer as does a non-lysogen, and a Lambda lysogen plates Lula at the same titer as does a non-lysogen (Fig. 2A). Lambdas with immunities of 21 and 434 lambdoid phages also plate on Lula lysogens (Fig. 2A). Lula/Lambda double lysogens can be easily generated (Fig. 2B), indicating that the two phages are different not only in immunities, but also in attachment sites.

To gain insights into Lula's horizontal spread in the laboratory setting, we decided to compare its general temperate phage characteristics in relation to its infectivity and productivity to those of Lambda [13], which is not known to spread under laboratory conditions. We noticed that the lysis/lysogeny decision for phage attacking cells grown in a rich medium, which is heavily skewed towards lysis for Lambda, is skewed more towards lysogeny for Lula. This is most easily seen in the simple streak test for lysogeny, which unequivocally reveals Lambda lysogens, but does not work for Lula (Fig. 2B). In both cases, the cells growing after crossing the phage line are all lysogens, but their titer is very low if the phage is Lambda (resulting in discontinuity of the streak), yet it is barely decreased if the phage is Lula (Fig. 2B). This preference for lysogeny should make instances of Lula infections on plates inconspicuous, helping Lula to silently spread among laboratory strains.

In contrast to Lambda lysogens, Lula lysogens are resistant to T4 (Fig. 2C). This should be inconsequential in the laboratory though as, unlike in the wild, infections with T4-type lytic phages rarely threaten survival of laboratory strains. On the other hand, since P1-mediated transduction seems to be the major route of Lula spread in laboratory, Lula's behavior during this common laboratory procedure could promote its horizontal spread. Indeed, we found that, in contrast to Lambda lysogens, Lula lysogens interfere with P1 development, decreasing the P1 titer by an order of magnitude (Fig. 2D). In addition, the titer of both Lula and Lambda virions in cultures of lysogens is increased by an order of magnitude by P1 infection (Fig. 2D). This double trick allows Lula to enrich P1 lysates with its own virions, so instead of the expected 100∶1 ratio (based on normal P1 development and the regular Lula titer around lysogens), P1 lysates prepared on Lula lysogens have 1∶1 ratio of P1 to Lula (Fig. 2D). We also noticed a 10^4^-times higher titer for Lula over Lambda in the mock P1 lysates made on the corresponding lysogens, which we will return to later.

### One-step growth protocol and UV-induction: Lambda vs. Lula

**Figure 3:**
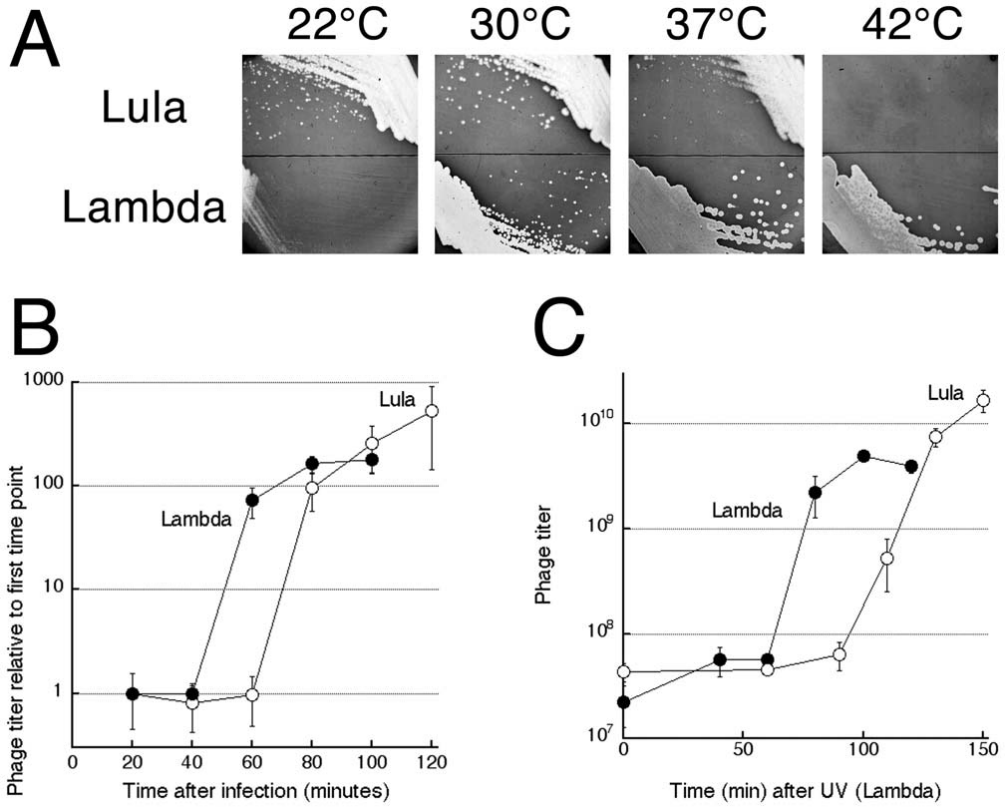
Lula shows an inverted temperature gradient compared to Lambda. **A.** The optimal temperatures for lytic growth of Lula versus Lambda. The corresponding phages from high titer lysates were streaked on a freshly-poured lawn of susceptible bacteria (AB1157). **B.** One-step growth at 37°C on AB1157. The data points are means of 3–5 independent measurements, done on different days, ± SEM. **C.** UV-induction of lytic development from a lysogen at 37°C. Lula lysogen, EL103; Lambda lysogen, EL104. The data points are means of three (for Lambda) or four (for Lula) independent measurements, done on different days, ± SEM.

While Lambda is known to prefer higher temperatures, in our hands showing the fastest growth at 42°C (gauged by the plaque size) (Fig. 3A), Lula's optimum temperature is 30°C, as it displays severe inhibition at 37°C and no growth at 42°C (Fig. 3A). In fact, Lula showed considerable growth at 22°C, the temperature at which Lambda's development almost stops (Fig. 3A). To quantify the slower growth of Lula versus Lambda at 37°C, we used the one-step growth protocol [14] to determine 1) the length of the phage infection cycle; 2) the phage burst size. In LB at 37°C, Lambda-infected cells start lysing at 45 minutes, the phage reaching 200X original titer ( =  the burst size) in 80 minutes, while Lula-infected cells start lysing at 60 minutes, the phage reaching 500X original titer at 120 minutes (Fig. 3B). Therefore, at 37°C, Lula's lytic development is slow; however, since Lula lysogens are more sensitive to DNA damage, we expected that Lula's lytic *induction* from lysogen would be comparable to that of Lambda or even faster.

However, kinetics of the lytic induction of a lysogen by UV at 37°C was also faster for Lambda, which started lysing by 60 minutes, while a Lula lysogen started lysing only after 90 minutes (Fig. 3C). Thus, because of the significantly longer infection cycle, combined with comparable-to-lambda burst size, at 37°C Lula comes out as a much less productive lytic phage compared even to the notoriously inefficient Lambda. For example, Lambda and Lula reach burst size of 100X in, correspondingly, 60 and 80 minutes. If subsequent infections have the same timing and burst size, in 240 minutes Lambda will produce 10^8^ particles (four infection cycles), while Lula will produce only 10^6^ particles (three infection cycles). The lower temperature optimum for lytic development means that at 37°C, a typical growth temperature for *E. coli* in the laboratory, Lula's lytic infection of a culture is so slow that it would be frequently overlooked — a clear benefit for cryptic spreading.

### Lula virions are more stable in cultures of lysogens

**Figure 4:**
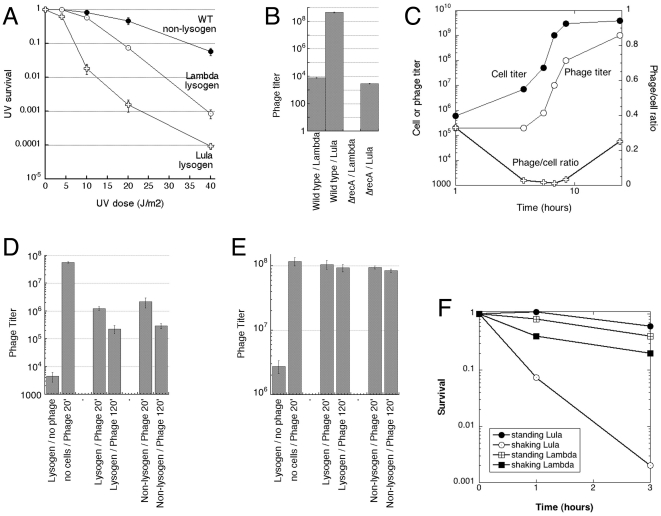
Testing possible explanations for high phage titer in cultures of Lula lysogens. **A.** UV-sensitivity of lysogens. Strains: wild type non-lysogen, AB1157; Lambda lysogen, EL104; Lula lysogen, EL103. The data points are means of 3–4 independent measurements, done on different days, ± SEM. **B.** Phage titer in saturated cultures of wild type (EL103 and EL104) and Δ*recA* lysogens (Lula, EL105; Lambda, EL106). The data points are means of five independent measurements, done on the same day, ± SEM. **C.** Cell titer versus Lula titer in the culture of a Lula lysogen (EL103) as a test for possible quorum sensing. Note that X axis is in log scale, as the left Y axis, but the right Y axis (for the lower curve) is linear. **D.** Stability of Lambda in saturated cultures of Lambda lysogen (EL104) and non-lysogen (AB1157). The data points are means of 3 independent measurements, done on different days, ± SEM. **E.** Stability of Lula in saturated cultures of Lula lysogen (EL103) and non-lysogen (AB1157). The data points are means of 3 independent measurements, done on different days, ± SEM. **F.** Stability of Lambda and Lula against aerosolation. The loss of phage titer in either stationary or rapidly shaking (to generate frothing) 1% NaCl suspensions was determined at two time points.

Lula lysogens were dramatically more sensitive to UV irradiation compared to Lambda lysogens, displaying both a shorter resistance shoulder and a steeper viability decline (Fig. 4A) and generally behaving like a moderately-defective DNA repair mutant. Perhaps Lula prophage goes lytic more easily because Lula's repressor is cleaved more readily by the RecA filaments than Lambda's repressor. Assuming the stability of Lula and Lambda virions is similar, the ease of induction should make cultures of Lula lysogens carry a heavier load of the culture virions compared to Lambda lysogens, which was already evident with “mock P1-lysates”(Fig. 2D). Indeed, in the supernatants of saturated cultures, Lula lysogen of wild type *E. coli* has an almost five orders of magnitude higher titer of the infectious phage particles than Lambda wild type lysogen (Fig. 4B). Surprised by this dramatic difference, we checked whether Lula lysogens are also induced independently of RecA, by measuring culture titers in Δ*recA* lysogens. As expected, Lambda Δ*recA* lysogen had no detectable culture phage titer (Fig. 4B). In contrast, Lula Δ*recA* lysogen still had some phage in the supernatant, although its titer was five orders of magnitude lower than in the wild type host (Fig. 4B). Thus, Lula induction is mostly RecA-dependent, like in Lambda, but, unlike Lambda, Lula may be triggered by even the transient RecA polymerization at spontaneous DNA lesions.

Another possibility explaining the high titer of culture phage in Lula lysogens was “quorum sensing”: a hypothetical stimulation of the lytic induction of the prophage by high titers of the extracellular cognate phage. This idea envisioned relatively low phage titers at low host cell densities, combined with a disproportionate increase once the cell titer grows and the culture titer of Lula raises over certain levels. We tested these predictions by measuring the culture titer of Lula at different cell densities (Fig. 4C, the top two curves), but did not find significant differences over the course of culture growth to saturation, although the phage/cell ratio *did* drop 30-fold in fast-growing cultures and then rebounded as the cultures became saturated (Fig. 4C, the bottom curve).

The most straightforward explanation for the high phage titers in Lula lysogen cultures would be higher stability of phage virions in cultures of the corresponding lysogens. To test this idea, we grew saturated cultures of Lambda or Lula lysogens, washed the cells to remove the resident phage, resuspended these stationary cells in spent sterile LB and then added similar high titer of the corresponding phage to the cognate lysogens. Upon further incubation, we found that Lambda, as expected, lost titer rapidly in the presence of cells that are immune to it, most likely because of attempted infection into the lysogens (Fig. 4D). In contrast, Lula's titer was stable in the presence of its lysogen, supposedly because Lula lysogens were resistant against superinfection (Fig. 4E). However, when we substituted susceptible non-lysogens (also stationary cells) for lysogens, we observed exactly the same loss of titer in Lambda and the same resistance in Lula (Fig. 4D and E), indicating that resistance to superinfection (although not ruled out) is not an explanation in this case, but Lula simply does not infect stationary cells, while Lambda does. We conclude that both the ease of lytic induction and the inability of Lula to infect stationary cells are responsible for the much higher phage titers in the saturated cultures of Lula lysogens compared to the Lambda ones. We consider the extremely high phage titer in cultures of Lula lysogens as one of the major contributors to this phage's ability to spread in the laboratory, because it makes it so infectious.

### Aerosol stability

Man-made aerosols must be the major route of horizontal spread in the laboratory for any type of microorganism, as aerosols are generated by many laboratory procedures, especially those that involve shaking and dispensing liquids [15], [16]. Since our original Lula detection was actually triggered by a periodic lysis of non-lysogen cultures processed in parallel with cultures of Lula lysogens, Lula is most certainly also transmitted through aerosol droplets. However, aerosol droplets pose two major challenges as contamination spread vehicles: 1) the greatly increased surface-to-volume ratio in aerosols magnifies the protein denaturation effect of surface tension; 2) rapid drying of aerosol droplets leads to desiccation. Hence, in order to be able to spread via aerosolation, Lula should be able to survive either surface tension or desiccation.

We found both Lambda and Lula to be quite resilient to desiccation, if dried on a plastic surface from a high titer stock: after an overnight incubation at room temperature the remaining titer in the dried spot was still around 10% of the original titer for either phage. This result was in line with the published data, as bacteriophages generally lose 90–95% of the original titer soon after drying, but then are able to maintain the remaining 5–10% of the titer for months, if kept dry under optimal conditions [17], [18], [19]. This surprising resistance of phages to drying is a natural adaptation and an obvious explanation for the bizarre phage aerosol stability curve, when the variable is the humidity of the chamber into which phage-containing aerosols are sprayed. The phages turn out to be quite resistant to aerosols when the humidity is either low (when the droplets dry fast) or high (when small droplets grow and consolidate into bigger ones), but are sensitive to aerosols when humidity is around 50%, when the droplets become smaller and then are stabilized [20], [21], [22], [23]. Surface tension in aerosol droplets denature phage proteins, literally bursting virions apart [24].

Since we did not have proper equipment to run aerosolation experiments under varied humidity, we tested the stability of Lula and Lambda to surface tension via rapid shaking in liquid, which introduces multiple small bubbles in the phage suspension and is generally compatible with aerosolation in its killing effect on phages [22], [25], [26]. We found that both Lula and Lambda lose titer if shaken in 1% NaCl, with Lula, actually, being more sensitive, losing almost three orders of magnitude in three hours (Fig. 4F). However, we also found that, when shaken in LB, both phages are perfectly stable (not shown), which is also consistent with the previous data on factors (like tryptone) protecting against surface tension [21], [22], [25]. We conclude that, although both resistance to desiccation and to surface tension in broth apparently contribute to Lula's infectivity, neither parameter is responsible for the difference in the potency of horizontal spread between Lula and Lambda.

### The mechanism of lysis trigger

**Figure 5:**
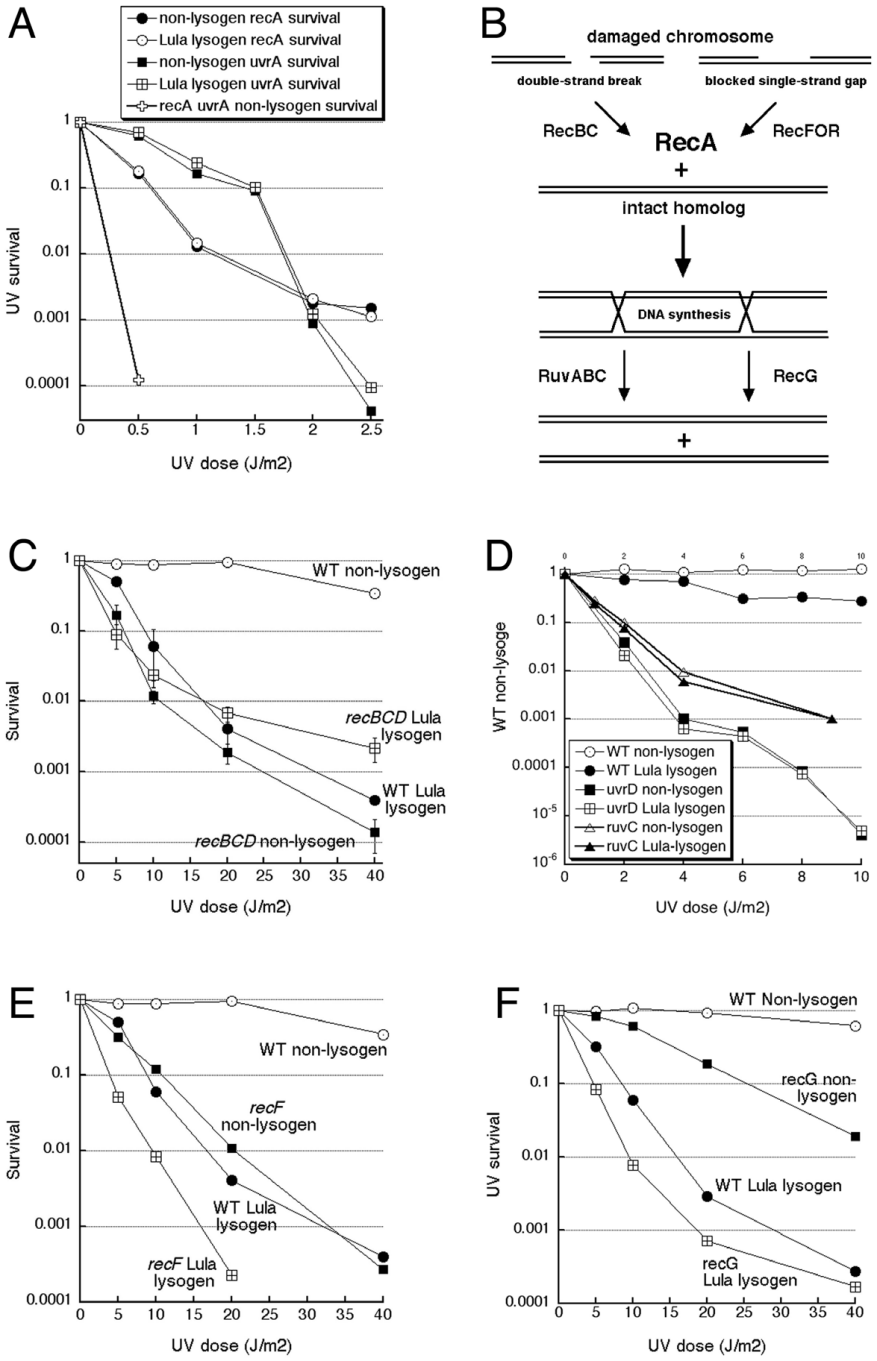
Epistatic analysis of Lula prophage versus DNA repair mutants sensitivity to UV irradiation. **A.** Interaction of Lula prophage with the *recA* and *uvrA* defects. Strains: LAP2, 3, 11, 12 and 15. **B.** A scheme of the recombinational repair pathways. **C.** Interaction of Lula prophage with the *recBCD* defect. Strains: GR523, AK147, LAP1 and LAP4. **D.** Interaction of Lula prophage with the *uvrD* and *ruvC* defects. Strains: GR523, LAP1, 7, 8, 13 and 14. **E.** Interaction of Lula prophage with the *recF* defect. Strains: GR523, LAP1, 9 and 10. **F.** Interaction of Lula prophage with the *recG* defect. Strains: GR523, LAP1, 5 and 6.

In order to get insights into why, in response to DNA damage, Lula decides to go lytic so early compared to Lambda, we measured the prophage-caused UV irradiation sensitivity (taking it as a measure of degree of the lytic induction) in hosts carrying various DNA repair defects (Fig. 5). In essence, we did a classic epistatic analysis [27], treating the prophage-induced sensitivity as if it were another DNA repair defect, but interpreting our data in the following way. If we observed *epistasis* (the prophage does not increase DNA damage sensitivity of the mutant), we took it to mean that the prophage killing depends on the corresponding function and is, therefore, eliminated in its absence. In other words, this function acts to generate the inducing signal. *Additivity* (the total killing effect is between the sum and the product of the individual killing effects due to the mutation and the prophage) would mean that the mutant killing and the prophage induction happen independently of each other, by separate mechanisms. Finally, *synergism* (the total killing effect is significantly higher than the product of the two individual killing effects) would mean that the defect of the mutant enhances the prophage induction. In other words, the corresponding function acts to reduce the inducing signal.

UV damage to the chromosomal DNA in *E. coli* is mended by two major repair pathways: nucleotide excision repair and recombinational repair [28], [29]. The corresponding critical activities in the two DNA repair systems are UvrA and RecA. Both the *uvrA* and *recA* mutants are extremely sensitive to UV irradiation [30], [31], and making them Lula lysogens does not increase this sensitivity further (for example, to the level of the extra sensitive *uvrA recA* double mutant [30], [31]) (Fig. 5A). In the case of the *recA* mutant, the observed epistasis could have reflected Lula's requirement for RecA filamentation to react to DNA damage; however, since it is unlikely that Lula requires UvrA-initiated excision for induction, we think that epistasis in both the *recA* and *uvrA* cases reflects the tightness of regulation of Lula's repression. Indeed, while Lula's induction begins sharply around UV dose of 5 J/m^2^ (Fig. 4A and 5CE), at this dose the *recA* or *uvrA* mutants are already dead, while Lula is, apparently, still tightly repressed below 5 J/m^2^ of UV.

Recombinational repair consists of two early pathways, RecBC and RecFOR, leading to the recombination intermediate, catalyzed by RecA, and branching into two late pathways, RuvABC and RecG (Fig. 5B) [32], [33]. Interaction of Lula induction with the two early pathways was especially interesting, since, as we have shown, Lula's induction requires RecA filamentation in response to DNA damage, and RecA filamentation on ssDNA is licensed by the early activities — RecBCD acting on double-strand ends or RecFOR acting on persistent single-strand gaps [32], [33]. Conveniently, and in contrast to the exquisitely sensitive *recA* and *uvrA* mutants, Lula lysogen has approximately the same intermediate UV-sensitivity as the *recBC* or *recF* mutants (Fig. 5C and E), so the additional effects in the double mutants should be clearly seen. We found that *recBCD* mutant Lula lysogen has the same sensitivity as the wildtype Lula lysogen or *recBCD* mutant at lower UV doses, suggesting that Lula-inducing signal is generated by the RecBCD-catalyzed double-strand end processing. Since the *recBCD* mutant Lula lysogen becomes more resistant over both wildtype Lula lysogen or the *recBCD* non-lysogen at higher UV doses (Fig. 5C), this means that 1) some Lula functions ameliorate the DNA repair defect of the *recBCD* mutants; 2) RecBCD enzyme contributes to cell killing during UV-induction of Lula lysogen.

In the UV damage repair, the late recombinational repair function RuvABC (Fig. 5B) acts mostly in the RecBCD pathway [34]. Consistent with the *recBCD* result, Lula prophage also does not increase UV sensitivity of the *ruvC* mutant (Fig. 5D). Another mutation which is epistatic to Lula for sensitivity to DNA damage is *uvrD*, a late defect in the UvrA-initiated nucleotide excision repair (Fig. 5D) [28]. Again, epistasis in this case may be due to high sensitivity of the *ruvABC* and *uvrD* mutants to UV, so that Lula remains mostly repressed when these mutants are already mostly dead (see above).

In contrast to the *recBCD* result, the *recF*-defective Lula lysogen is significantly more sensitive to UV than either wild type Lula lysogen or the *recF* non-lysogen (Fig. 5E), suggesting that Lula is induced by UV independently of the RecFOR pathway that assembles RecA filaments on the persistent single-strand gaps. In UV damage repair, the late recombinational repair function RecG (Fig. 5B) acts mostly in the RecFOR pathway [35]. Consistent with the *recF* interactions, Lula prophage greatly increases the UV sensitivity of the *recG* mutant (Fig. 5F), again suggesting independence of Lula's induction of the repair of persistent single-strand gaps. In summary, our epistatic analysis of UV-sensitivity caused by Lula versus various DNA damage repair defects points out to double-strand DNA breaks as the proximal triggers of Lula lytic induction. It is remarkable that, at the doses of UV that induce Lula, all these double-strand breaks are still repairable, and the cells are killed only because they carry the prophage. More importantly for the enhancement of horizontal spread in the laboratory, induction of the Lula lytic development reacts to spontaneous and frequent chromosomal lesions, because spontaneous double-strand breaks happen almost every generation [32]. In contrast, massive chromosomal damage of the type that induces prophage Lambda happens in the laboratory setting only in controlled circumstances of the DNA damaging treatments, not easily compatible with cryptic horizontal spread.

### Lula is identical to phi80

Sequencing of the Lula genome confirmed both its length (46,150 bp) and that it indeed had not been published before (E.R. and A.K., unpublished). However, several genes of Lula matched exactly the few sequenced genes of phi80, a lambdoid phage isolated by Matsushiro in 1961 [36] and widely used in the 1970s and 1980s in phage studies [37]. Eventually we tracked down a completed, but never published, phi80 genome sequence to the Blattner laboratory at the University of Wisconsin (Guy Plunkett III, personal communication). Comparison of the two genomes, — Lula from Illinois versus phi80 from Wisconsin, — showed that they were identical, so the cross-contaminating prophage turned out to be the original phi80. Thus, the two known idiosyncrasies of phi80, — growth inhibition at high temperature [38], [39] and the inability to infect non-growing cells [37], — turned out to be the factors facilitating Lula's horizontal spread in the laboratory.

## Discussion

The ability to secure resources allows organisms to be productive and prosper in natural environments, but how an organism prospers in the laboratory environment, where both the access to resources and their available amount for reproduction are tightly controlled by humans, was intriguing. After finding a contaminating prophage, Lula, in *E. coli* strains from several sources, we investigated its characteristics that allow it to colonize the laboratory strains without human authorization, spreading horizontally without being noticed in one of the most restrictive environments. *A priori*, generic qualifications for cryptic horizontal spread in the laboratory environment should include: 1) stability against aerosolation/desiccation, as aerosols are likely to be the major horizontal spread mechanism in the laboratory; 2) either experimental material commensality or mimicry, to hide the non-sanctioned growth; 3) stealthy infectivity — efficient infection of diverse non-contaminated materials with a minimal subsequent evidence of contamination.

**Figure 6:**
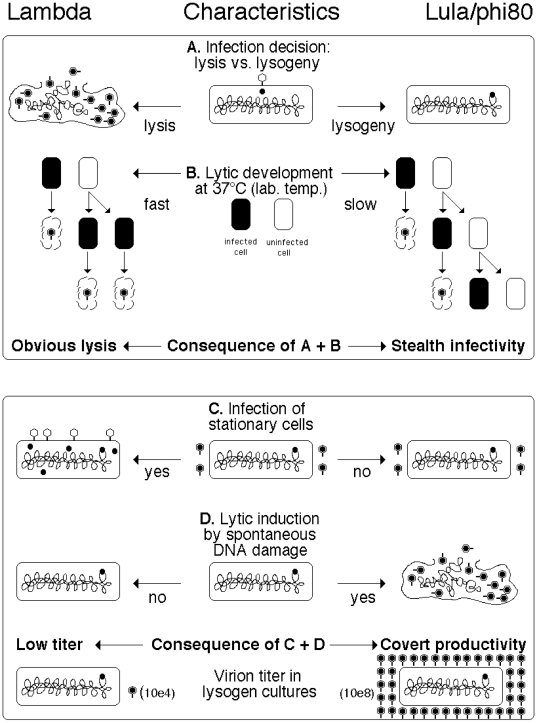
Characteristics of Lambda and Lula/phi80 contributing to their different levels of spread in laboratory environment.

Lula, which turned out to be phi80, is a temperate phage of *E. coli*, which, simply by the fact of being a phage, is reasonably resistant to both desiccation [17], [18], [19] and surface tension due to aerosolation/shaking/bubbling [20], [21], [22], [23], fulfilling qualification number 1. By being a prophage of the most common laboratory organism, *E. coli*, Lula/phi80 also fulfills qualification number 2 (experimental material commensality). But in both respects Lula/phi80 is not different from the well-characterized temperate phage Lambda, which is not known to spread in the laboratory. We found the following traits that, compared to analogous characteristics of Lambda, specifically adapt Lula/phi80 to survival via horizontal spreading in the laboratory environment by enhancing its stealthy infectivity (qualification number 3) (Fig. 6):

A. Preference for lysogeny over lysis upon initial infection, making Lula/phi80 a more temperate phage than Lambda (Fig. 6A). This preference allows Lula infection of both liquid cultures and colonies to remain inconspicuous and difficult to spot, compared with infection by lytic phages or even temperate phages like Lambda.

B. Slow lytic development at 37°C (Fig. 6B). The temperature sensitivity of Lula/phi80 makes it an almost non-lytic phage compared to Lambda at 37°C, the typical temperature of *E. coli* growth in the laboratory. Our success with catching Lula/phi80 was mostly due to the fact that the bulk of culture growth in this laboratory is done at 28°C — the temperature at which Lula grows much better than Lambda, revealing its lytic development.

C. Inability of Lula/phi80 to attach to stationary cells (Fig. 6C). The active metabolism requirement for Lula/phi80's attachment to cells [37] must have evolved in the wild, to increase Lula's chances of productive infection, but turned out to be critical for spreading among laboratory cultures, that are artificially cycled between rapid growth and stasis.

D. Ease of lytic induction from lysogeny by spontaneous chromosomal lesions (Fig. 6D). This contributes to the high culture titer of the phage, since Lula/phi80's specific sensitivity to double-strand breaks makes it inducible even by spontaneous DNA damage. It should be pointed out that the sensitivity of Lula/phi80 lysogens to DNA damage, in combination with our interest in DNA repair mutants, served as a critical juncture that made Lula/phi80's identification possible in the first place.

E. Active stance towards infection by other phages. We found that, in contrast to Lambda, Lula/phi80 lysogen completely prevents T4 development and interferes with P1 development, reducing P1 titer 10-fold. In parallel, Lula/phi80's own lytic development is mildly induced by P1 infection (Lambda does the same), so that P1 lysates coming from Lula/phi80 lysogens have an equal ratio of the two phages.

Remarkably, out of these five traits, only traits “A” (preference for lysogeny upon infection) and “B” (temperature sensitivity) should contribute to stealthy infectivity of Lula/phi80, while the other three traits reveal additional qualifications for survival via horizontal spread in the laboratory, which we, *a posteriori*, can identify as: 4) covert productivity — continuous production of the agent by the contaminated research material to the highest possible level which is still inconspicuous, achieved via crude synchronization of replication of the agent with the one of the research material; 5) stability against the distinct challenges of the laboratory environment (like survival in saturated cultures); 6) “protocol hitchhiking” — facilitated spread of the agent via common laboratory practices and protocols. Thus, trait “D” (hair-trigger lytic induction) fulfills the requirement number 4 for covert productivity, trait “C” (requirement of active metabolism of the host cells for Lula/phi80 attachment) fulfills the requirement number 5 for stability in the laboratory environment, while together traits “C”, “D” and “E” (lytic induction by P1 infection with simultaneous inhibition of P1 development) contribute to the high titer in cultures of Lula/phi80 lysogens and in P1 lysates, fulfilling the requirement number 6 (spread through hitchhiking on common laboratory practices — growing cultures to saturation, aerosol-generating liquid handling, P1 transduction). Specifically, the high culture/lysate virion loads (up to 10^9^ per ml) make Lula/phi80 infection possible by 1 pL (10^−9^ mL) aerosol droplets (roughly 10 µm in diameter). Various laboratory liquid-handling procedures generate such aerosol microdroplets [15], [16]. In contrast to Lula/phi80, other well-characterized temperate phages of *E. coli*, like Lambda, P1, P2 and Mu all have the low culture virion loads of 10^4^ per ml (this study, [40]), which makes them virtually non-infectious via the aerosol route.

In conclusion, our study of the principles of survival and reproduction in the laboratory environment via unauthorized horizontal spread using the temperate lambdoid *E. coli* bacteriophage Lula/phi80 revealed them as stealth (in this case, via commensality with the common laboratory material), stability (resistance in the laboratory protocols) and infectivity (via covert yet aggressive productivity and laboratory protocol hitchiking). These should be taken into considerations while reviewing good laboratory practices, as Lula/phi80, together with *Helacyton gartleri* (HeLa cells, recognized by Van Valen as a separate species adapted to laboratory spread [5]) may only represent a tip of the iceberg of cryptic laboratory dwellers, serving us with a warning that our control of the laboratory environment has limits that Life learned to break. Since Lula/phi80 has been around for almost 50 years, its silent spread likely affects a significant fraction of the accumulated *E. coli*-based experimental material. There is a lot of anecdotal evidence about phi80 contamination in the molecular biology lore, but none of it is published; the reason, perhaps, being primarily a social one, described by the Contribution Games (a cousin of infamous Prisoner's Dilemma) [41]. Such a broad phenomenon would be hard to suppress completely, though, but then mis-identification might have helped Lula/phi80 to escape attention. For example, since the superinfection immunity gene *cor* of phi80 prevents infection with a lytic T1 phage, because the two phages share the same receptor, FhuA (TonA) [42], [43], one wonders whether the ubiquitous contamination with “T1-like” phages of various BAC libraries [10], [11], [12] is, in fact, due to Lula/phi80. T1 is a lytic phage, — therefore its infections should be self-limiting and, therefore, easy to control. On the other hand, Lula/phi80 is a temperate phage, which is more consistent with the “carrier” status of contaminated bacterial clones. Fighting “T1 contamination” could be notoriously difficult, the cited reason being T1 resistance to desiccation [44]; however, since many bacteriophages, including lambda, Lula/phi80 and T1 are more-or-less resistant to desiccation ([17], [18], [19], this work), one wonders whether the actual contamination is coming from aerosols of phi80 lysogens. Parenthetically, it should be noted that the same *cor* gene of phi80, that qualifies it as “T1-like”, might have been responsible for the initial spread of the phi80 infection, as an alternative to *tonAB* resistance to T1 infection.

On the practical side, when dealing with cultures of Lula/phi80 lysogens, this phage's resistance to desiccation can be countered by UV-irradiation, P1 transductants should be recovered at 42°C or higher temperatures, while spreading through aerosols can be controlled via laminar hoods and filter pipette tips.

## Materials and Methods

### Strains, media and growth conditions

Bacterial strains used in this study are in Table 1. Various *uvr* and *rec* mutants were confirmed using their characteristic UV sensitivities. Bacteria were propagated on LB-agar plates. LB broth per 1 L contains: 10 g tryptone, 5 g yeast extract, 5 g NaCl, pH brought to 7.4 with 250 µl of 4M NaOH; LB agar contained 15 g agar per 1 liter of LB broth. TM buffer is 10 mM Tris-HCl pH 8.0, and 10 mM MgSO_4_. BBL agar contains 10 g BBL trypticase, 5 g NaCl, 250 µl 4 M NaOH, and 15 g agar per liter.

**Table 1:** Bacterial strains used in this study.

Name	Relevant genotype	Source/derivation/reference
**Previous studies**		
AB1157	wild type	[45]
AK147	Δ*recBCD*::*kan*	[46]
GR523	Hfr *thi*	[47]
GS1481	*ruvC64*::*kan*	[48]
JC10287	Δ(*recA-srlR*)*304*	[49]
MO (λi^21^)		Jeff Gardner
MO (λi^21^)(phi80)(λ*vir* ^R^)		Jeff Gardner
N2731	*recG258*::Tn*10*(mini-*kan*)	[35]
WA576	*recF400*::Tn*5*	[50]
**This study**		
AK4	Δ(*srlR-recA*)*306*::Tn*10*	laboratory collection
AK44	AB1157 *uvrA6* malE::Tn*10*(*kan*)	laboratory collection
AK111	Δ*uvrD288*::*kan*	laboratory collection
EL103	wild type (Lula)	lysogenized AB1157
EL104	wild type (λ)	lysogenized AB1157
EL105	Δ(*recA-srlR*)*304* (Lula)	lysogenized JC10287
EL106	Δ(*recA-srlR*)*304* (λ)	lysogenized JC10287
LAP1	Δ*ligB*::*cat* (Lula)	lysogenized GR523 Δ*ligB*
LAP2	*uvrA6* (*kan*)	GR523 x P1 AK44
LAP3	*uvrA6* (*kan*) Δ*ligB*::*cat* (Lula)	LAP1 x P1 AK44
LAP4	Δ*recBCD*::*kan* Δ*ligB*::*cat* (Lula)	LAP1 x P1 AK147*
LAP5	*recG258*::Tn*10*(mini-*kan*)	GR523 x P1 N2731
LAP6	*recG258*::Tn*10*(mini-*kan*) Δ*ligB*::*cat* (Lula)	LAP1 x P1 N2731
LAP7	*ruvC64*::*kan*	GR523 x P1 GS1481
LAP8	*ruvC64*::*kan* Δ*ligB*::*cat* (Lula)	LAP1 x P1 GS1481
LAP9	*recF400*::Tn*5*	GR523 x P1 WA576
LAP10	*recF400*::Tn*5* Δ*ligB*::*cat* (Lula)	LAP1 x P1 WA576
LAP11	Δ(*srlR-recA*)*306*::Tn*10*	GR523 x P1 AK4**
LAP12	Δ(*srlR-recA*)*306*::Tn*10* Δ*ligB*::*cat* (Lula)	LAP1 x P1 AK4**
LAP13	Δ*uvrD288*::*kan*	GR523 x P1 AK111
LAP14	Δ*uvrD288*::*kan* Δ*ligB*::*cat* (Lula)	LAP1 x P1 AK111
LAP15	*uvrA6* (*kan*) Δ(*srlR-recA*)*306*::Tn*10*	LAP2 x P1 AK4**

*complemented with a *recBCD*+ plasmid.

**complemented with a *recA*+ plasmid.

### Detection of Lula lysogens

A suspected strain was grown to saturation in LB, and 0.4–1 ml of culture was centrifuged for 4 minutes. 10 µl of the supernatant was spotted on a BBL plate containing 150 µl of a saturated culture of AB1157 or another sensitive strain mixed with 4 ml top agar (equal parts TM buffer and BBL agar) and incubated for 1 hour at 30°C before spotting. If a P1 lysate was being tested, 330 µl of 1 M sodium citrate was added to the top agar, to prevent P1 infection. After overnight incubation, the supernatants from Lula-carrying strains or Lula-contaminated lysates formed large clear zones in the lawn of cells. For this reason, antibiotics - particularly kanamycin - were omitted from the overnight culture to prevent false positives. If kanamycin was present, then the supernatant was diluted ten-fold in TM before spotting.

### Isolating phage stock

A single isolated plaque grown on an AB1157 lawn in BBL agar was punched out of the plate using a capillary tube and expelled into 1 ml TM buffer. The phages were dispersed into the buffer over 1–2 hours with occasional brief vortexing. 30 µl of the eluate was combined with 300 µl plating cell culture (AB1157 grown to OD_600_ = 0.5 in LB, pelleted and resuspended in TM buffer) at 37°C for 15 minutes. 3 ml of top BBL/TM agar was then added, and the contents of the tube were poured on a BBL plate. After 6–7 hours incubation at 37°C, when the lawn had cleared, the plate was overlaid with 5 ml of TM buffer overnight at room temperature. In the morning, the TM was collected, and a fresh layer of 4 ml TM was added. After additional 8 hours, the remainder of the TM was collected, the combined eluate was centrifuged for 10 minutes at 8,500 g, transferred to a fresh glass tube, and 50 µl of chloroform was added to kill surviving bacteria.

### Isolation of Lula DNA from virions

450 µl of phage stock (prepared as above, but using 1% agarose instead of 1.5% agar in the BBL plate) was combined with 50 µl of 10% SDS and briefly vortexed. DNA was extracted consecutively with 500 µl phenol, then with 500 µl phenol/chloroform 1∶1 mixture, and finally with 500 µl chloroform (with 5 minute centrifugations at 16,000 g after every extraction to separate the phases). The final aqueous phase was ethanol-precipitated twice and dissolved in 100 µl of TE buffer. If the phage DNA was to be used to make a probe, the phage stock was treated with 2U of DNaseI (NEB) for 15 minutes at 37°C before extraction, to remove *E. coli* DNA.

### One-step growth

AB1157 cells were subcultured to OD_600_ = 0.2, and 1 ml was placed on ice for 15 minutes. Phage stocks in either in LB or TM buffer were combined with the cells on ice for 15 minutes at the multiplicity of infection of approximately 10. After adsorption, cells were incubated at 37°C for 15–20 minutes, washed, and resuspended in 1 ml LB. The culture was grown at 37°C, and 100 µl aliquots were serially diluted in 1% saline at the indicated times. 10 µl of each dilution for each time point was spotted on a lawn of AB1157 in BBL top agar.

### UV induction

Saturated cultures of lysogens were diluted 100-fold into fresh medium, grown to OD_600_ = 0.2, and cells from 1 ml of the cultures were collected by centrifugation and resuspended in 1 ml of a 1% NaCl, 0.02% TritonX-100 solution. The removal of growth medium was necessary because the tryptophan in LB protects cells from UV; the detergent allowed the suspension to spread evenly. The mixture was placed on a rimmed cover of a Petri dish and irradiated with 40 J/m^2^. 900 µl was retrieved, cells were collected by centrifugation, resuspended in 900 µl LB and grown at 37°C. At the indicated times, 100 µl aliquots were removed and serially diluted ten-fold in LB. 10 µl of each dilution was spotted on BBL supplemented with 0.1% SDS for the cell titer and to a BBL plate with an AB1157 top agar lawn for phage titer.

### Quantitative survival after various DNA damaging treatments

In all cases, the protocol would go through the same basic pre-treatment and post-treatment steps. Pre-treatment included inoculating cultures with individual colonies, shaking them overnight at 28°C, diluting in the morning 100-fold and continued shaking at 28°C until they reached OD_600_ 0.2 - 0.3. Post-treatment included taking aliquots at the indicated times, serially diluting them in 1% NaCl and spotting by 10 µl onto LB agar plates. Plates were incubated overnight at 28°C. The survivors on the deepest dilutions were counted under the stereo microscope while they were still small to yield a titer at specific treatment doses; those were then normalized to the original titer, to yield the survival curve.

Treatments with specific DNA-damaging agents (while shaking at 28°C in the growth medium) were as follows:

Hydrogen peroxide: final concentration of 2 mM, the treatment time was fixed for 20 minutes.

Nalidixic acid: 400 µl of culture were mixed with 1.6 ml of warm LB containing 40 µl of a 5 mg/ml nalidixic acid stock (the stock was made just before the treatment by dissolving nalidixic acid crystals in 25 mM NaOH), doses were regulated by time of treatment.

MMS: final concentration of 0.3%, doses were regulated by time of treatment.

UV-irradiation protocol was different. The rapidly-growing cultures were serially diluted in 1% NaCl, the six dilutions were spotted by 10 µl onto LB or BBL agar square plates in six rows (one strain per 36-position plate, six spots of the same dilution per row) and allowed to dry. The plates were irradiated with a UV gradient perpendicular to the dilution gradient and incubated overnight in the dark at 28°C.

### T4 infection

Cultures of AB1157 and its Lambda or Lula lysogens were grown to an approximate OD_600_ = 0.3 and combined with 3.5 ml LB top agar on top of an LB plate. After drying for 20 minutes, serial dilutions of T4 in TM were spotted on the lawn and the plate was incubated overnight at 30°C.

### Desiccation

1 or 10 µl of phages suspended in LB was placed in the bottom of a 1.5 ml microcentrifuge tube and dried overnight under a fume hood. The next day, 100 µl LB was added to the tube, which was then occasionally vortexed for at least 10 minutes, and the redissolved phage was serially diluted in LB. The titer of the redissolved phage was determined by spotting on a BBL/TM top agar lawn relative to the original titer of the untreated phage.

### Resistance to fast shaking

Lambda or Lula stocks were added to large glass tubes containing 4 ml of either LB or 1% NaCl and either shaken vigorously at 250 rpm at an approximately 18° angle from the horizontal or allowed to stand without movement at 30°C for 1 hour, 3 hours, or overnight. Phages were serially diluted in TM buffer and titered on a BBL/TM lawn.

### Stability of phages in saturated cultures

Cultures of AB1157 or its Lambda and Lula lysogens were grown to saturation. Cells were washed twice in LB to remove excess phages from the supernatant and resuspended in the equal volume of spent LB (sterilized supernatant of overnight cultures). Approximately 1×10^8^ phages were added to the cultures, which were incubated at 37°C for 20 minutes and 2 hours. After the cells were pelleted, the supernatant was serially diluted and titered on BBL/TM top agar lawn. As controls, an equivalent volume of spent LB was added to cultures instead of phage (with later determining the phage titer), and the titer of the phages was also taken in the absence of cells, to determine its resistance to spent LB.

### Interaction with P1 infection

Lambda or Lula lysogens were subcultured in 3 ml LB with 0.2% glucose and grown to approximately 5×10^8^ cells/ml. The cultures were supplemented with CaCl_2_ (to 5 mM) and infected for 20 minutes at 30°C with either 50 µl of P1vir (2.5×10^6^ pfu) or spent LB (AB1157 grown to saturation with the cells removed by filtration). 8 ml of top agar (LB with 7.5 g agar/1 L) was mixed with the cells and spread over two LB plates supplemented with 5 mM CaCl_2_ and 0.2% glucose. After 14–17 hours incubation at 30°C, the top agar layer was collected, crushed and combined with 3 ml LB and 500 µl chloroform for at least 10 minutes. The cell-containing agar was removed by centrifugation at 10,000 rpm (8,000 g) for 20 minutes, and the phage-containing supernatant saved with a drop of chloroform.

To titer P1, AB1157 was grown to OD_600_ = 0.4 and resuspended in LB containing 5 mM CaCl_2_ and 10 mM MgSO_4_. 100 µl of cells was incubated at 37°C for 15–20 minutes with 100 µl of the lysate diluted in LB. 500 µl of LB top agar containing 5 mM CaCl_2_ was added to the infected cells and the 700 µl was dispensed onto one quadrant of a Petri dish containing LB agar. The plate was incubated overnight, and plaques were counted the following day. To prevent Lula plaques, the plate was incubated at 42°C. To prevent Lambda plaques, a Lambda lysogen was used, and the plate was incubated at 37°C. To titer Lula in the presence of P1, serial dilutions were spotted on a BBL/TM top agar lawn containing 80 mM NaCit. To titer Lambda, serial dilutions were spotted on a BBL/TM top agar lawn containing 16 mM NaCit and 5 mM MgSO_4_.
